# Supplementary material for: Immune subset-committed proliferating cells populate the human foetal intestine throughout the second trimester of gestation
Source: Nat Commun. 2023 Mar 10;14:1318. doi: 10.1038/s41467-023-37052-4 (PMC10006174; doi:10.1038/s41467-023-37052-4)
Supplement: Supplementary file 1 — Supplementary Information [file 41467_2023_37052_MOESM1_ESM.pdf]

Supplementary information

Immune subset-committed proliferating cells populate the human foetal intestine throughout the second trimester of gestation

Nannan Guo[1], Na Li[1,2], Li Jia[1], Qinyue Jiang[1], Mette Schreurs[1], Vincent van Unen[1,3], Susana M. Chuva de Sousa Lopes[4], Alexandra A. Vloemans[1], Jeroen Eggermont[5], Boudewijn Lelieveldt[5], Frank J.T. Staal[1], Noel F. C. C. de Miranda[6], M. Fernanda Pascutti[1*], Frits Koning[1 *]

[1]Department of Immunology, Leiden University Medical Center, Leiden, Netherlands

[2]State Key Laboratory of Zoonotic Diseases, Institute of Zoonoses, College of Veterinary Medicine, Jilin University, Changchun, China

[3]Institute for Immunity, Transplantation and Infection, Stanford University, Stanford, CA, United States

[4]Department of Anatomy, Leiden University Medical Center, Leiden, Netherlands

[5]Department of Radiology, Leiden University Medical Center, Leiden, The Netherlands

[6]Department of Pathology, Leiden University Medical Center, Leiden, Netherlands

[*] **Equal contribution**

Frits Koning email: F.Koning@lumc.nl

M. Fernanda Pascutti email: M.F.Pascutti@lumc.nl

Supplementary Figures

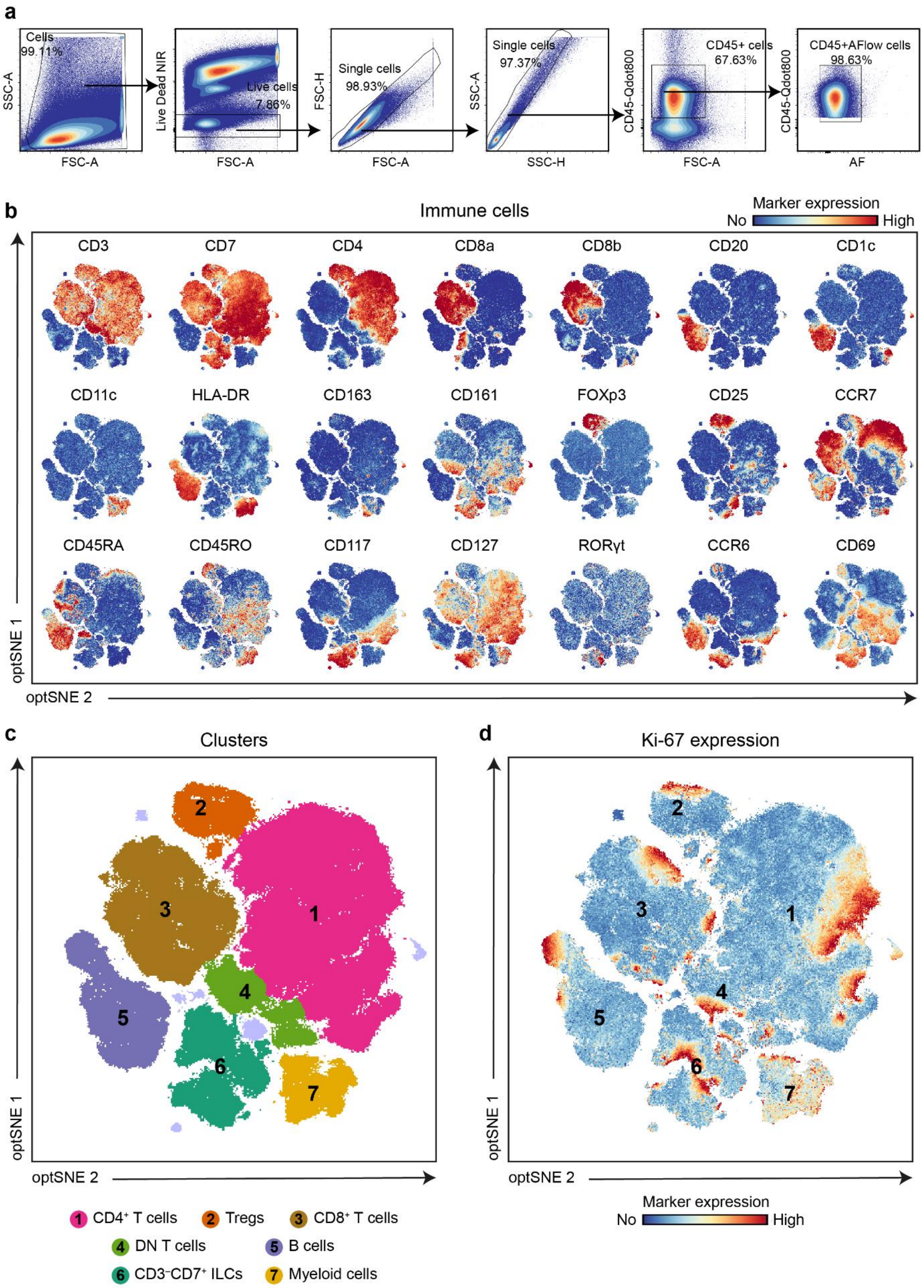

Supplementary Fig. 1 Identification of Ki-67$^+$ cells within all immune subsets in the human fetal intestine

(a) The gate strategy of immune cells from spectral flow cytometry datasets. **(b)** A collective optSNE was performed on CD45$^+$ immune cells from 7 human fetal intestinal samples in a second experiment. Each dot represents a single cell. In total, 5×10^5 CD45$^+$ immune cells were analyzed by OMIQ. Colors represent relative expression of indicated immune markers. **(c)** Based on the relative marker expression profile, each immune lineage was identified and color-coded. **(d)** Display of the Ki-67 expression within the CD45$^+$ immune cells. Colors represent relative expression of Ki-67.

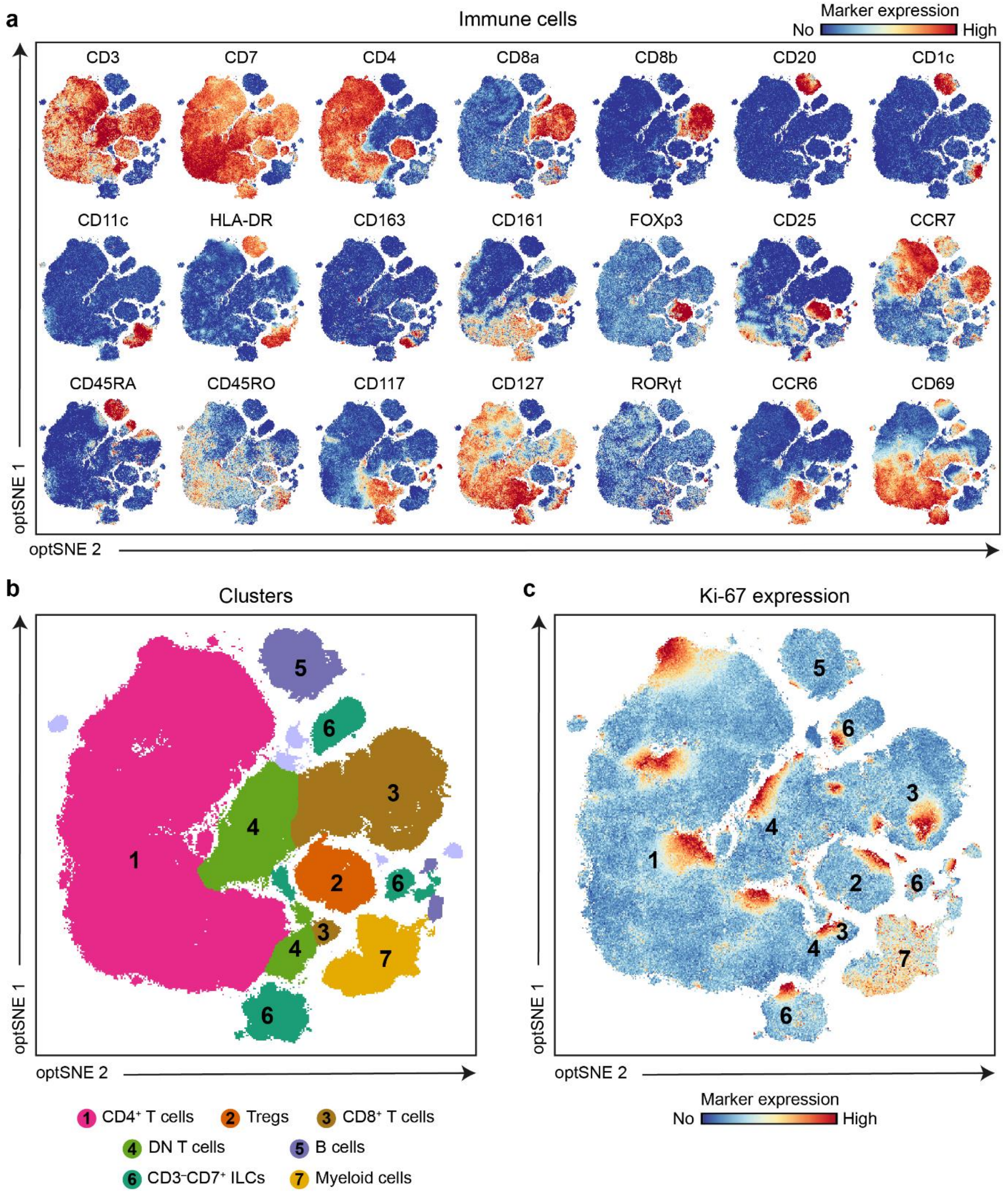

Supplementary Fig. 2 Identification of Ki-67⁺ cells within all immune subsets in the human fetal intestine

(a) A collective optSNE was performed on CD45⁺ immune cells from 9 human fetal intestinal samples in a third experiment. Each dot represents a single cell. In total, 1.02×10^6 CD45⁺ immune cells were analyzed by OMIQ. Colors represent relative expression of indicated immune markers. **(b)** Based on the relative marker expression profile, each immune lineage was identified and color-coded. **(c)**

Display of the Ki-67 expression within the $CD45^+$ immune cells. Colors represent relative expression of Ki-67.

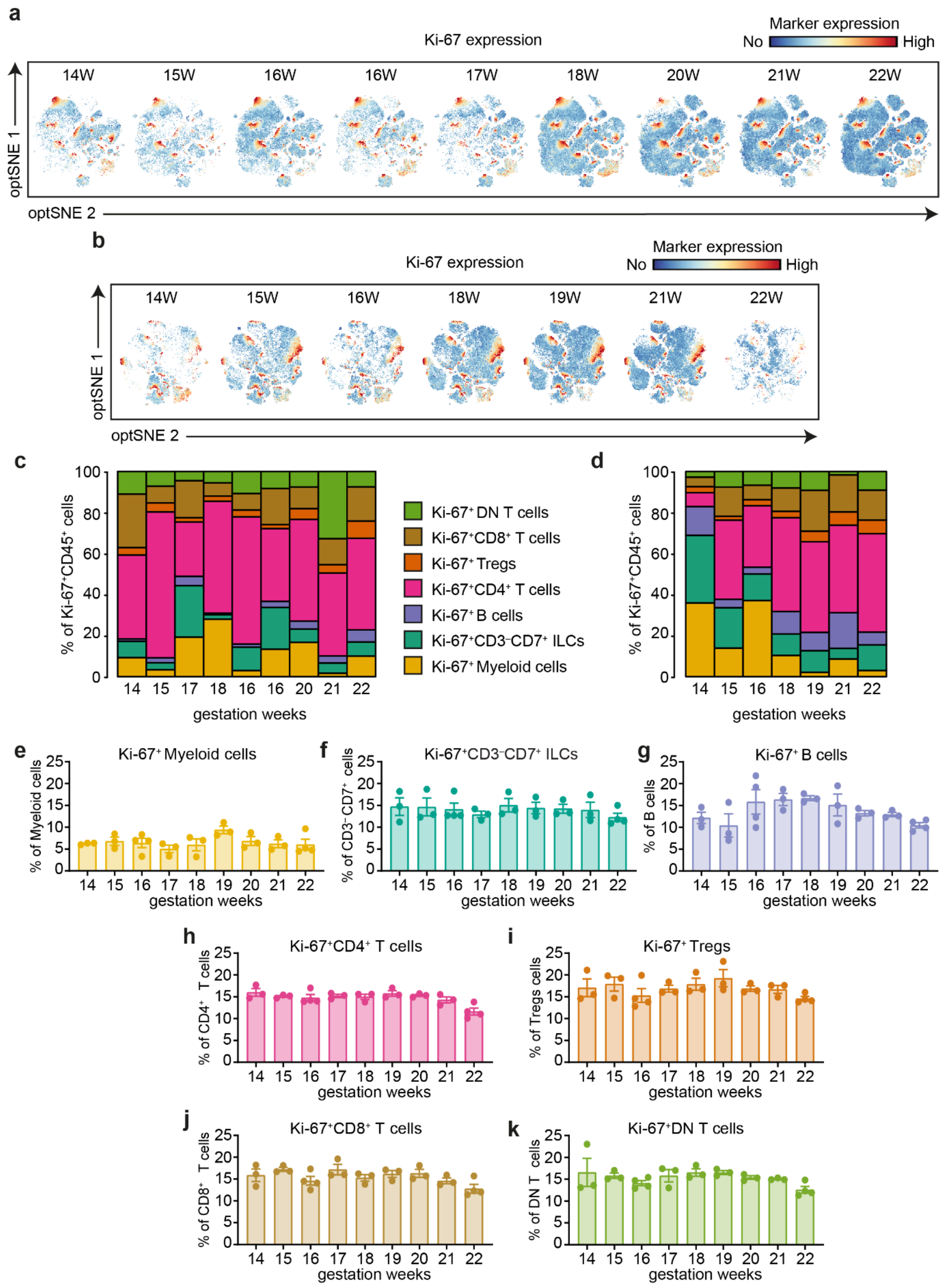

Supplementary Fig. 3 Presence of Ki-67^{+} cells throughout gestational week 14 to 22

The presence of Ki-67^{+} cells in fetal intestinal samples from gestational week 14 through 22 was analyzed in two additional experiments. **(a)** Display of the Ki-67 expression in the optSNE plots of the

individual fetal intestinal samples (n = 9) from gestational week 14 through 22 in a second experiment. Colors represent different level of Ki-67 expression. **(b)** Display of the Ki-67 expression in the optSNE plots of the individual fetal intestinal samples (n = 7) from gestational week 14 through 22 in a third experiment. Colors represent different level of Ki-67 expression. **(c-d)** Overview of the distribution of Ki-67$^+$ cells in the indicated immune lineages from gestational week 14 through 22 in the additional two experiments. Colors indicate different immune subsets. **(e-k)** Overview of the percentages of Ki-67$^+$ cells within each immune subset in all human fetal intestine samples analyzed (n = 28). Error bars indicate mean ± s.e.m.

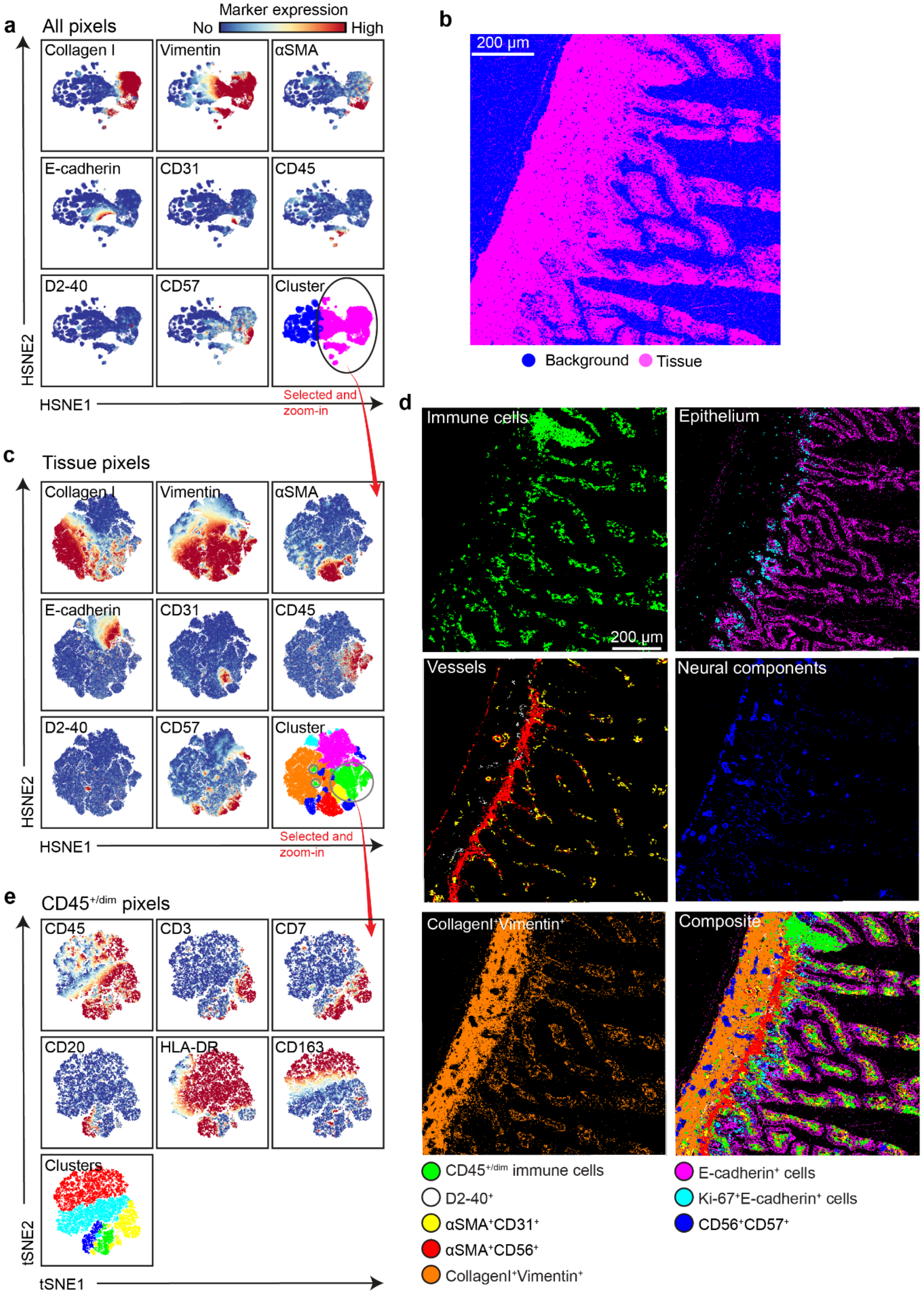

Supplementary Fig. 4 The workflow of pixel analysis for IMC data by *Cytosplore imaging*.

(a) An HSNE was performed on pixels from a ROI (1000 µm × 1000 µm) of a human fetal intestine sample. Each dot represents a pixel (1 μm^2). Colors represent the relative expression of indicated markers. **(b)** Based on marker expression profile, the structural markers, CD45 (a) and DNA staining (not shown), the tissue pixels (colored pink) were distinguished from the non-tissue pixels (colored blue), and both were projected to the image viewer. **(c)** The tissue pixels were selected as indicated and a new higher resolution embedding was generated. Colors represent relative expression of indicated markers. **(d)** Based on marker expression profile (c), in this level, eight distinct clusters of pixels were identified and color coded. These clusters represented immune cells ($CD45^{+/dim}$), 2 populations of epithelial cells ($E\text{-cadherin}^{+}Ki\text{-}67^{+}$ and $E\text{-cadherin}^{+}Ki\text{-}67^{-}$), endothelial cells ($\alpha SMA^{+}CD31^{+}$), smooth muscle cells ($\alpha SMA^{+}CD31^{-}$), lymphatic endothelial cells ($D2\text{-}40^{+}$), neural components ($CD57^{+}CD56^{+/-}$) and extracellular matrix of the basement membrane (Collagen $I^{+}Vimentin^{+}$). **(e)** A further tSNE analysis of the $CD45^{+/dim}$ pixels identified the various immune cell clusters. Colors represent relative expression of indicated immune markers.

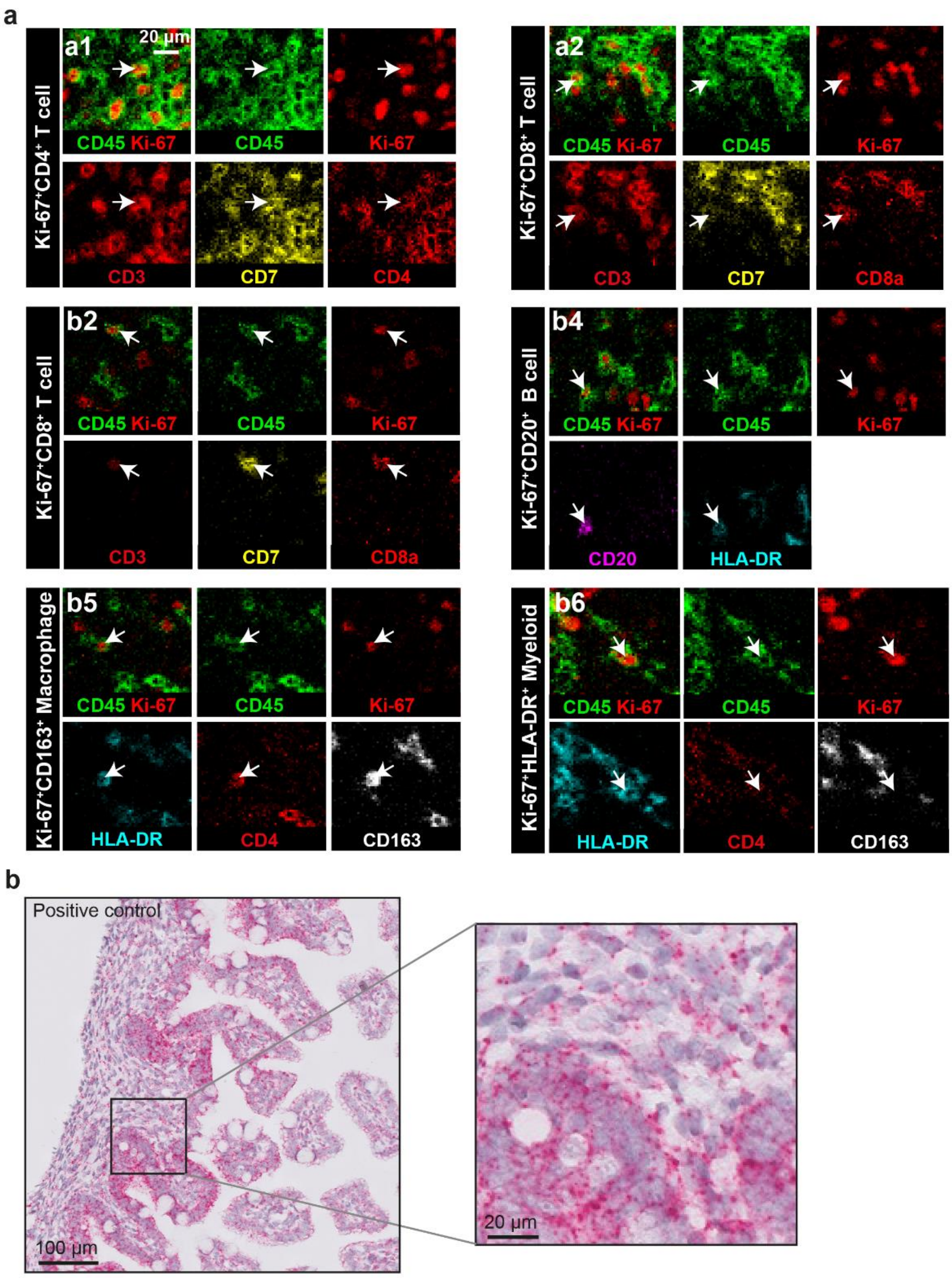

Supplementary Fig. 5 Visualization of Ki-67$^+$ cells by IMC and RNAscope assays on human fetal intestines.

(a) The combination of Ki-67 and immune cell markers was used to visualize **(a1 in Fig. 6a)** Ki-67$^+$CD4$^+$ T cells, **(a2 and b2 in Fig. 6a, b)** Ki-67$^+$CD8$^+$ T cells, **(b4 in Fig. 6b)** Ki-67$^+$CD20$^+$ B cells, **(b5 in Fig. 6b)** Ki-67$^+$CD163$^+$ macrophage, and **(b6 in Fig. 6b)** Ki-67$^+$Lin$^-$HLA-DR$^+$ myeloid cells in human fetal intestinal samples as indicated by white arrows. (b) The image of *PPIB* probe as positive control in RNAscope assays on the human fetal intestine.

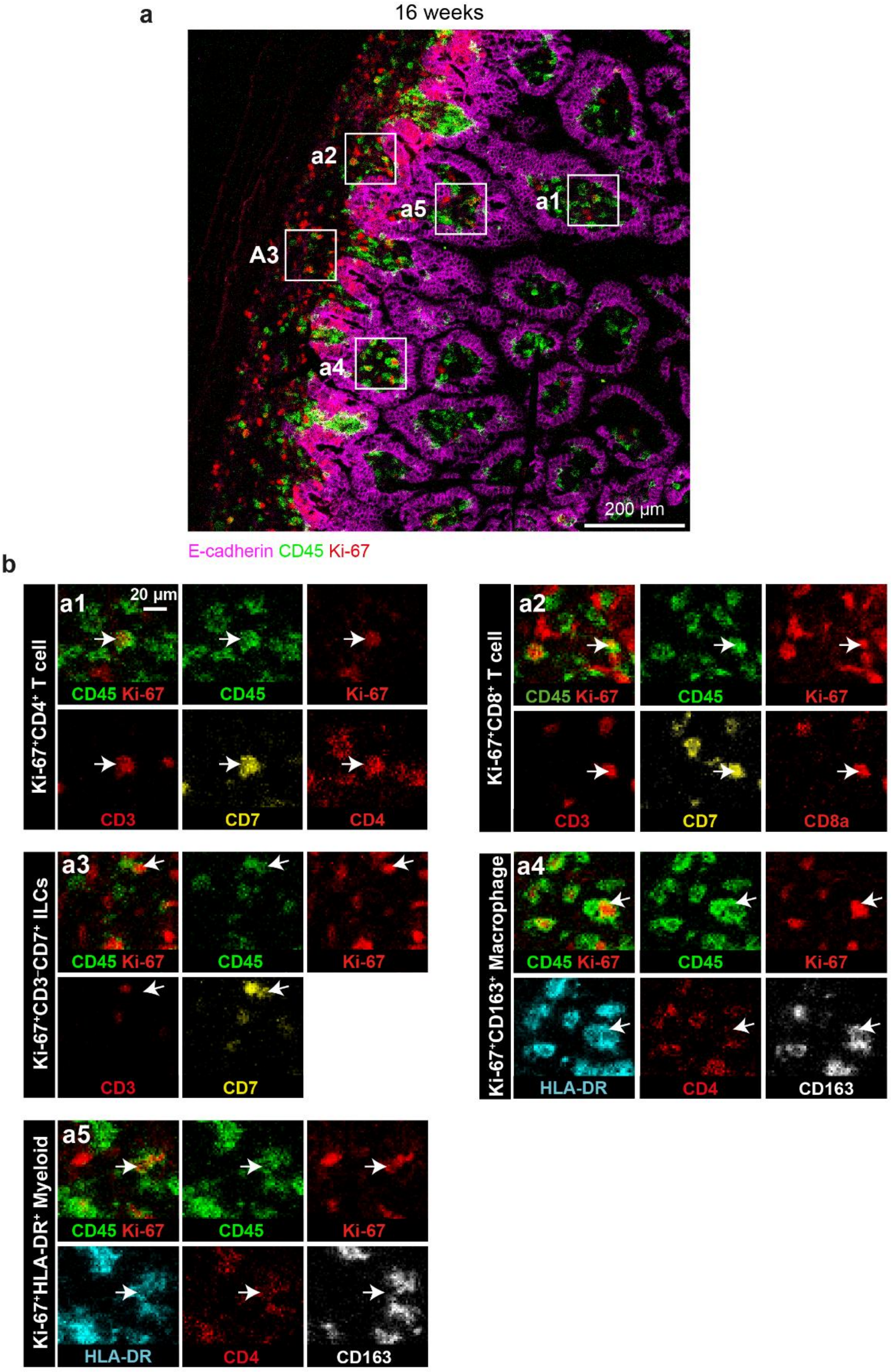

Supplementary Fig. 6 Visualization of Ki-67$^+$ cells by IMC in a human fetal intestine from gestational week 16

(a) Visualization of Ki-67$^+$CD45$^+$ cells by staining of Ki-67 and CD45. Scale bar, 200 µm. **(b)** The combination of Ki-67 and immune lineage markers visualized **(a1)** Ki-67$^+$CD4$^+$ T cells, **(a2)** Ki-67$^+$CD8$^+$ T

cells, **(a3)** Ki-67$^+$CD3$^-$CD7$^+$ ILCs, **(a4)** Ki-67$^+$CD163$^+$ macrophage, and **(a5)** Ki-67$^+$Lin$^-$HLA-DR$^+$ myeloid cells in *situ*. White arrow indicated cells of interest. Scale bar, 20 μm**.**

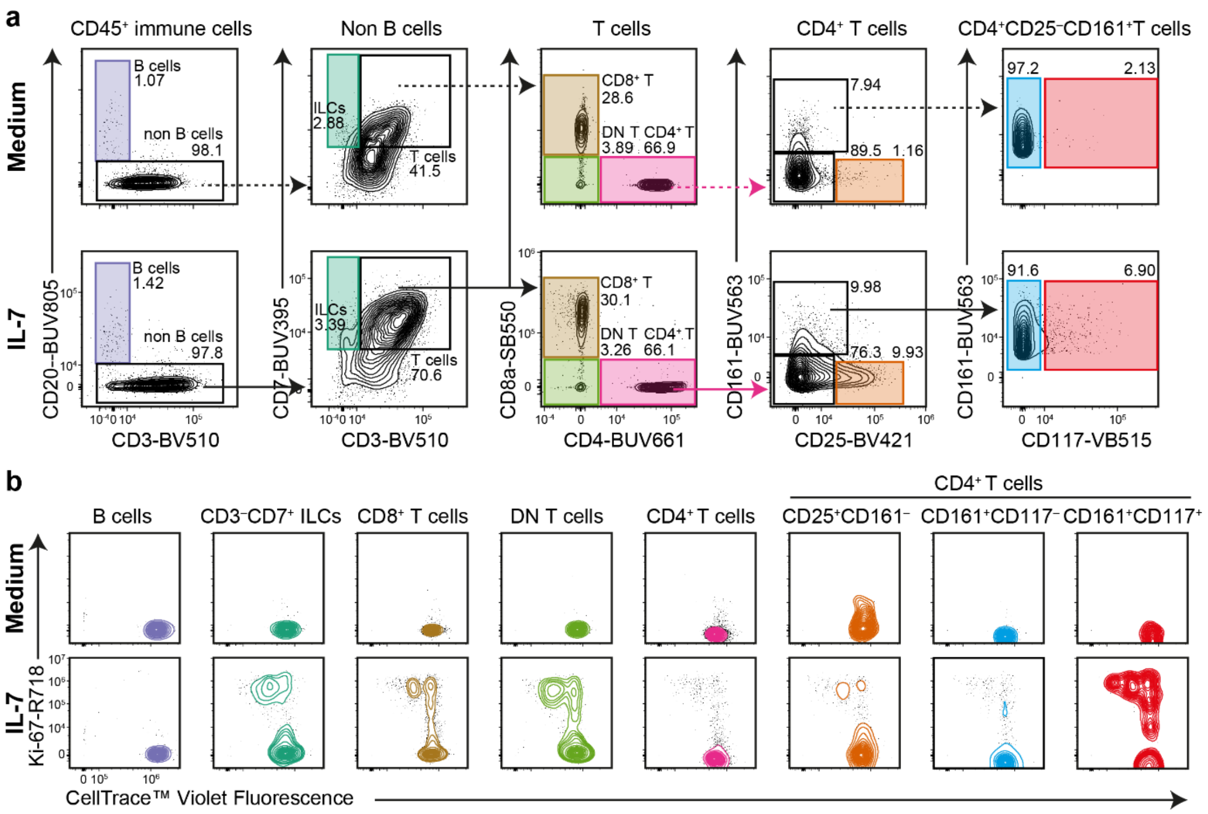

Supplementary Fig. 7 Proliferation assays of Ki-67+ cells in each immune subsets from the PBMCs sample

(a) Biaxial plots showing the identification of investigated immune subsets by Cytek Aurora flow cytometry in the PBMCs in medium and medium with IL-7. Colors indicate different immune subsets. **(b)** Biaxial plots showing expression of Ki-67 versus CellTrace™ Violet dye dilution on the indicated immune subsets in the PBMCs sample cultured in medium and medium with IL-7. Colors indicate different immune subsets.

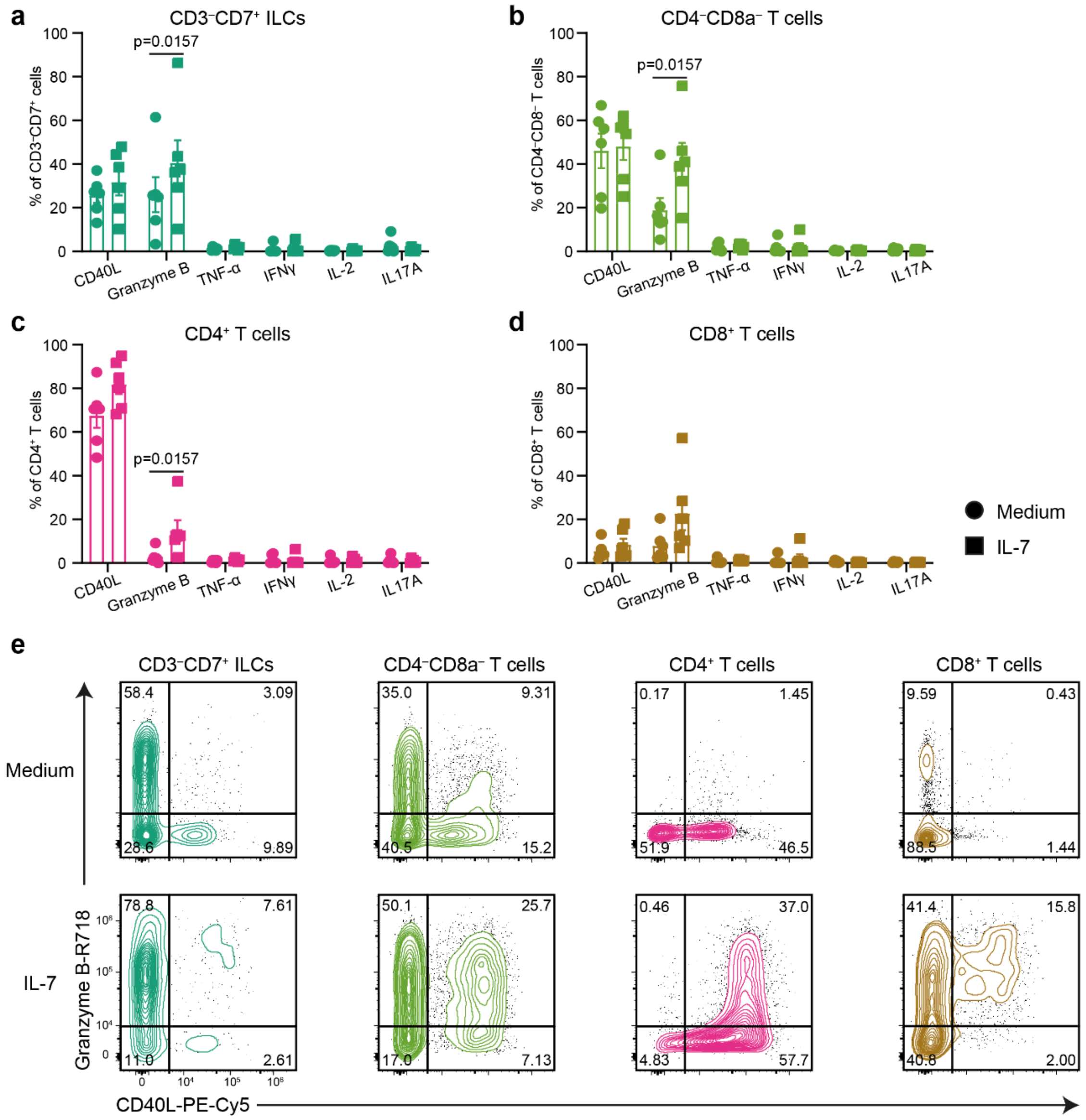

Supplementary Fig. 8 Functional profiling of fetal intestinal cells

(a-d) Fetal intestinal cells were cultured in the absence or presence IL-7 for 48 hours followed by flow cytometric analysis of the expression of CD40L, granzyme B, TNF-α, IFNγ , IL-2 , and IL-17A in $CD3^{-}CD7^{+}$ ILCs and T cells ($CD4^{-}CD8^{-}$ T, $CD4^{+}$ T and $CD8^{+}$ T). In total, four independent experiments were performed (n=6). Error bars indicate mean ± s.e.m. *$P < 0.05$, Wilcoxon signed-rank test with two-tailed for comparisons. **(e)** Intracellular expression of granzyme B and CD40L was determined for $CD3^{-}$ $CD7^{+}$ ILCs and T cells ($CD4^{-}CD8^{-}$ T, $CD4^{+}$ T and $CD8^{+}$ T) by flow cytometry in both culture conditions. The biaxial plots show data from one representative experiment.

Supplementary Tables

Supplementary Table 1 Single-cell suspension spectral flow cytometry antibody panel.

	Antigen	Tag	Clone	Supplier	Cat.	Dilution
1	CCR6	BB700	11A9	BD Biosciences	566478	40
2	CCR7	Spark NIR 685	G043H7	Biolegend	353257	25
3	CD117	VioBright 515	REA787	MACS	130-111-674	200
4	CD11c	PE/Cy7	3.9	Biolegend	301608	100
5	CD127	BV711	A019D5	Biolegend	351327	25
6	CD161	BUV563	HP-3G10	BD Biosciences	749223	25
7	CD163	BV650	GHI/61	BD Biosciences	563888	25
8	CD1c	SB436	L161	Invitrogen	62-0015-41	25
9	CD20	BV805	2H7	BD Biosciences	612906	50
10	CD25	BV421	BC96	Biolegend	302630	50
11	CD3	BV510	UCHT1	Biolegend	300448	100
12	CD34	PE/CF594	581	BD Biosciences	562383	50
13	CD4	CF568	SK3	CyTek	R7-20041	200
14	CD45	QD800	HI30	Invitrogen	Q10156	100
15	CD45R0	BV570	UCHL1	Biolegend	304225	200
16	CD45RA	PerCP	HI100	Biolegend	304155	200
17	CD69	APC/R700	FN50	BD Biosciences	565155	50
18	CD7	BUV395	M-T701	BD Biosciences	565979	100
19	CD8a	Spark Blue 550	SK1	Biolegend	344759	200
20	CD8b	BUV496	2ST8.5H7	BD Biosciences	749837	200
21	FoxP3	APC	PCH101	Invitrogen	17-4776-42	25
22	HLA-DR	BV480	G46-6	BD Biosciences	566154	200
23	Ki67	BV605	Ki-67	Biolegend	350521	25
24	RORgt	PE	Q21-559	BD Biosciences	563081	25
25	Viability	L/D near IR	-	Invitrogen	L34976	1000
26	FC Block	-	-	Biolegend	422302	20

Supplementary Table 2 Imaging mass cytometry antibody panel on human fetal intestine from gestational week 21.

	Antigen	Tag	Clone	Supplier	Cat.	Dilution
1	CD45	89Y	HI30	FLM	3089003B	1/50
2	CD69	144Nd	FN50	FLM	3144018B	1/50
3	CD4	145Nd	RPA-T4	FLM	3145001B	1/50
4	CD8a	146Nd	RPA-T8	FLM	3146001B	1/50
5	Collagen I	147Sm	polyclonal	Millipore	AB758	1/100
6	α-SMA	148Nd	1A4	CST	CST 5685BF	1/200
7	E-cadherin	150Nd	24 E 10	CST	CST 3195BF	1/50
8	CD123	151Eu	6H6	FLM	3151001B	1/50
9	CD7	153Eu	CD7-6B7	FLM	3153014B	1/100
10	CD163	154Sm	GHI/61	FLM	3154007B	1/100
11	CD103	155Gd	EPR4166	Abcam	ab221210	1/50
12	CD122	158Gd	TU27	BioL	339015	1/25
13	CD68	159Tb	KP1	FLM	3159035D	1/100
14	CD5	160Gd	UCHT2	BioL	300627	1/25
15	CD20	161Dy	H1	FLM	3161029D	1/50
16	CD11c	162Dy	Bu15	FLM	3162005B	1/50
17	CD45	163Dy	D9M81	CST	13917BF	1/100
18	CD161	164Dy	HP-3G10	FLM	3164009B	1/50
19	Ki-67	166Er	D3B5	CST	CST 9129BF	1/200
20	CD27	167Er	O323	FLM	3167002B	1/50
21	HLA-DR	168Er	L243	BioL	307651	1/800
22	CD45RA	169Tm	HI100	FLM	3169008B	1/100
23	CD3	170Er	UCHT1	FLM	3170001B	1/100
24	CD28	171Yb	CD28.2	BioL	302937	1/50
25	CD38	172Yb	HIT2	FLM	3172007B	1/50
26	CD45RO	173Yb	UCHL1	BioL	304239	1/50
27	Vimentin	175Lu	D21H3	CST	CST 5741BF	1/200
28	CD56	176Yb	NCAM16.2	FLM	3176008B	1/100
Fluidigm (Flui), Cell Signaling Technology (CST) and Biolegend (BioL)						

Supplementary Table 3 Imaging mass cytometry antibody panel on human fetal intestine from gestational week 21.

	Antigen	Tag	Clone	Supplier	Cat.	Dilution
1	CD45	89Y	HI30	Flui	3089003B	1/50
2	D2-40	115In	D2-40	BioL	916606	1/50
3	FOXp3	142Nd	D608R	CST	12653BF	1/50
4	CD69	144Nd	FN50	Flui	3144018B	1/50
5	CD4	145Nd	RPA-T4	Flui	3145001B	1/50
6	CD8a	146Nd	RPA-T8	Flui	3146001B	1/50
7	Collagen I	147Sm	Polyclonal	Millipore	AB758	1/100
8	α-SMA	148Nd	1A4	CST	CST5685BF	1/200
9	CD31	149Sm	8 9C2	CST	CST3528BF	1/100
10	E-Cadherin	150Nd	24 E 10	CST	CST3195BF	1/50
11	CD123	151Eu	6H6	Flui	3151001B	1/50
12	CD7	153Eu	CD7-6B7	Flui	3153014B	1/100
13	CD163	154Sm	GHI/61	Flui	3154007B	1/100
14	CD103	155Gd	EPR4166	Abcam	ab221210	1/50
15	CD127	156Gd	R34.34	Beckman	18LIQ494	1/50
16	CD122	158Gd	TU27	BioL	339015	1/25
17	CD68	159Tb	KP1	Flui	3159035D	1/200
18	CD5	160Gd	UCHT2	BioL	300627	1/25
19	CD20	161Dy	H1	Flui	3161029D	1/50
20	CD11c	162Dy	Bu15	Flui	3162005B	1/50
21	CD45	163Dy	D9M81	CST	13917BF	1/200
22	CD161	164Dy	HP-3G10	Flui	3164009B	1/50
23	CD117	165Ho	104D2	BioL	313202	1/50
24	Ki-67	166Er	D3B5	CST	CST 9129BF	1/200
25	CD27	167Er	O323	Flui	3167002B	1/50
26	HLA-DR	168Er	L243	BIoL	307651	1/800
27	CD45RA	169Tm	HI100	Flui	3169008B	1/100
28	CD3	170Er	UCHT1	Flui	3170001B	1/100
29	CD28	171Yb	CD28.2	BioL	302937	1/50
30	CD38	172Yb	HIT2	Flui	3172007B	1/100
31	CD45RO	173Yb	UCHL1	BioL	304239	1/50
32	CD57	174Yb	HNK-1/Leu-7	Abcam	Ab212403	1/100
33	Vimentin	175Lu	D21H3	CST	CST5741BF	1/200
34	CD56	176Yb	NCAM16.2	Flui	3176008B	1/50
Fluidigm (Flui), Cell Signaling Technology (CST) and Biolegend (BioL)						

Supplementary Table 4 Imaging mass cytometry antibody panel on human fetal intestine from gestational week 16 to 19.

	Antigen	Tag	Clone	Supplier	Cat.	Dilution
1	CD45	89Y	HI30	Flui	3089003B	50
2	D2-40	115In	D2-40	BioL	916606	50
3	FOXp3	142Nd	D608R	CST	12653BF	100
4	CD69	144Nd	FN50	Flui	3144018B	50
5	CD4	145Nd	RPA-T4	Flui	3145001B	50
6	CD8a	146Nd	RPA-T8	Flui	3146001B	50
7	Collagen I	147Sm	polyclonal	Millipore	AB758	100
8	CD34	148Nd	QBEND/10	Thermo	MA1-10202	50
9	CD31	149Sm	8 9C2	CST	CST3528BF	100
10	E-cadherin	150Nd	24 E 10	CST	CST 3195BF	50
11	CD123	151Eu	6H6	Flui	3151001B	50
12	CD141	152Sm	Phx-01	BioL	902102	50
13	CD7	153Eu	CD7-6B7	Flui	3153014B	100
14	CD163	154Sm	GHI/61	Flui	3154007B	100
15	CD103	155Gd	EPR4166	Abcam	ab221210	50
16	CD127	156Gd	R34.34	Beckman	18LIQ494	50
17	CD68	159Tb	KP1	Flui	3159035D	200
18	CD20	161Dy	H1	Flui	3161029D	50
19	CD11c	162Dy	Bu15	Flui	3162005B	50
20	CD11c	162Dy	S-HCL-3	BioL	125602	50
21	CD161	164Dy	HP-3G10	Flui	3164009B	50
22	CD117	165Ho	104D2	BioL	313202	50
23	Ki-67	166Er	D3B5	CST	CST 9129BF	200
24	CD27	167Er	O323	Flui	3167002B	50
25	HLA-DR	168Er	L243	BIoL	307651	800
26	CD45RA	169Tm	HI100	Flui	3169008B	100
27	CD3	170Er	UCHT1	Flui	3170001B	100
28	CD1c	171Yb	L161	BioL	331501	50
29	CD38	172Yb	HIT2	Flui	3172007B	100
30	CD45RO	173Yb	UCHL1	BioL	304239	50
31	CD57	174Yb	HNK-1/Leu-7	Abcam	Ab212403	100
32	CD25	175Lu	24204.0	Thermo	MA5-23714	50
33	CD56	176Yb	NCAM16.2	Thermo	MA1-06801	50
34	α-SMA	194Pt	1A4	CST	56856BF	100
35	Vimentin	198Pt	D21H3	CST	5741BF	100
Fluidigm (Flui), Cell Signaling Technology (CST) and Biolegend (BioL)						

Supplementary Table 5 Target Genes and Probed Regions in RNAscope assays.

Gene Name	Accession Number	Probed Region (bp)
DapB	EF191515	414 - 862
PPIB	NM_000942.4	139 - 989
CXCL13	NM_006419.2	30 - 1186
IL7	NM_001199888.1	55 - 1482

Supplementary Table 6 The antibody panel of proliferation assays by spectrum flow cytometry.

	Antigen	Tag	Clone	Supplier	Cat.	Dilution
1	CD117	VioBright 515	REA787	MACS	130-111-674	200
2	CD11c	PE/Cy7	3.9	Biolegend	301608	100
3	CD127	BV711	A019D5	Biolegend	351327	25
4	CD161	BUV563	HP-3G10	BD Biosciences	749223	25
5	CD20	BUV805	2H7	BD Biosciences	612906	50
6	CD25	BV421	BC96	Biolegend	302630	50
7	CD3	BV510	UCHT1	Biolegend	300448	100
8	CD4	BUV661	SK3	BD Biosciences	612962	100
9	CD45	NovaBlue 610	2D1	Invitrogen	H005T02B05	50
10	CD7	BUV395	M-T701	BD Biosciences	565979	100
11	CD8a	SB550	SK1	Biolegend	344759	200
12	CD8b	BUV496	2ST8.5H7	BD Biosciences	749837	200
13	HLA-DR	BV750	L243	Biolegend	307671	50
14	Ki67	R718	B56	BD Biosciences	566963	100
15	Viability	L/D near IR	-	Invitrogen	L34976	1000
16	CellTrace™ Violet	CTV	-	Invitrogen	34557	4000
17	FC Block	-	-	Biolegend	422302	20

Supplementary Table 7 The antibody panel of functional profiles analysis by spectrum flow cytometry.

	Antigen	Tag	Clone	Supplier	Cat.	Dilution
1	CD117	VioBright 515	REA787	MACS	130-111-674	200
2	CCR7	Spark NIR 685	G043H7	Biolegend	353257	25
3	CD127	BV711	A019D5	Biolegend	351327	25
4	CD161	BUV563	HP-3G10	BD Biosciences	749223	25
5	CD20	BUV805	2H7	BD Biosciences	612906	50
6	CD25	BV421	BC96	Biolegend	302630	50
7	CD3	BV510	UCHT1	Biolegend	300448	100
8	CD4	BUV661	SK3	BD Biosciences	612962	100
9	CD45	NovaBlue 610	2D1	Invitrogen	H005T02B05	50
10	CD7	BUV395	M-T701	BD Biosciences	565979	100
11	CD8a	SB550	SK1	Biolegend	344759	200
12	CD8b	BUV496	2ST8.5H7	BD Biosciences	749837	200
13	CD45R0	BV570	UCHL1	Biolegend	304225	200
14	CD45RA	PerCP	HI100	Biolegend	304155	200
15	HLA-DR	BV750	L243	Biolegend	307671	50
16	Ki67	BV605	Ki-67	Biolegend	350521	25
17	Helios	PerCP/eFluor710	22F6	Thermo	46-9883-42	25
18	IL-17A	PE/Dazzle™ 594	BL168	Biolegend	512335	25
19	IFNg	BV750	RUO	BD Biosciences	566357	50
20	TNFa	PE/Cy7	MAb11	Thermo	25-7349-82	50
21	CD40L	PE/Cy5	24-31	Biolegend	310808	50
22	Granzyme B	R718	B56	BD Biosciences	566963	50
23	IL-2	PE	MQ1-17H12	BD Biosciences	560902	200
24	Viability	L/D near IR	-	Invitrogen	L34976	1000
25	CellTrace™ Violet	CTV	-	Invitrogen	34557	4000
26	FC Block	-	-	Biolegend	422302	20
