# Supplementary material for: Immune subset-committed proliferating cells populate the human foetal intestine throughout the second trimester of gestation
Source: Nat Commun. 2023 Mar 10;14:1318. doi: 10.1038/s41467-023-37052-4 (PMC10006174; doi:10.1038/s41467-023-37052-4)
Supplement: Supplementary file 3 — Reporting Summary [file 41467_2023_37052_MOESM3_ESM.pdf]

nature portfolio

Corresponding author(s): Frits Koning

Last updated by author(s): 2023/02/13

Reporting Summary

Nature Portfolio wishes to improve the reproducibility of the work that we publish. This form provides structure for consistency and transparency in reporting. For further information on Nature Portfolio policies, see our Editorial Policies and the Editorial Policy Checklist.

Statistics

For all statistical analyses, confirm that the following items are present in the figure legend, table legend, main text, or Methods section.

n/a	Confirmed	
☐	☒	The exact sample size (*n*) for each experimental group/condition, given as a discrete number and unit of measurement
☐	☒	A statement on whether measurements were taken from distinct samples or whether the same sample was measured repeatedly
☐	☒	The statistical test(s) used AND whether they are one- or two-sided<br>*Only common tests should be described solely by name; describe more complex techniques in the Methods section.*
☒	☐	A description of all covariates tested
☒	☐	A description of any assumptions or corrections, such as tests of normality and adjustment for multiple comparisons
☐	☒	A full description of the statistical parameters including central tendency (e.g. means) or other basic estimates (e.g. regression coefficient) AND variation (e.g. standard deviation) or associated estimates of uncertainty (e.g. confidence intervals)
☐	☒	For null hypothesis testing, the test statistic (e.g. *F*, *t*, *r*) with confidence intervals, effect sizes, degrees of freedom and *P* value noted<br>*Give P values as exact values whenever suitable.*
☒	☐	For Bayesian analysis, information on the choice of priors and Markov chain Monte Carlo settings
☒	☐	For hierarchical and complex designs, identification of the appropriate level for tests and full reporting of outcomes
☒	☐	Estimates of effect sizes (e.g. Cohen's *d*, Pearson's *r*), indicating how they were calculated

Our web collection on statistics for biologists contains articles on many of the points above.

Software and code

Policy information about availability of computer code

Data collection	Data for flow cytometry were acquired using an a 5-laser Cytek® Aurora cytometor (BD Biosciences). Data for imaging mass cytometry were acquired using a Hyperion imaging-mass cytometer (Fluidigm) at 1 µm resolution following the manufacturer's instructions.
Data analysis	The platform of OMIQ (https://www.omiq.ai/) was used for analysis of immunophenotypic studies by flow cytometry. Imaging mass cytometric data were analyzed by Fluidigm MCDTM viewer (v1.0.560.2), and Cytosplore imaging (v3.3.2). Cell Proliferation Assays by flow cytometry were analyzed by FlowJo software version 10.6 (Tree Star Inc). All statistics were analyzed using GraphPad Prism8 software.

For manuscripts utilizing custom algorithms or software that are central to the research but not yet described in published literature, software must be made available to editors and reviewers. We strongly encourage code deposition in a community repository (e.g. GitHub). See the Nature Portfolio guidelines for submitting code & software for further information.

Data

Policy information about availability of data

All manuscripts must include a data availability statement. This statement should provide the following information, where applicable:

- Accession codes, unique identifiers, or web links for publicly available datasets
- A description of any restrictions on data availability
- For clinical datasets or third party data, please ensure that the statement adheres to our policy

Source data are provided as a Source Data file. Source data are provided with this paper. Single-cell RNA-seq data is available via Gene Expression Omnibus

accession code GSE122846 (https://www.ncbi.nlm.nih.gov/geo/query/acc.cgi?acc=GSE122846). The flow cytometry data generated in this study have been deposited in are available via Flow Repository (http://flowrepository.org/id/FR-FCM-Z5MB). Imaging mass cytometry data are deposited at Mendeley Data (https://data.mendeley.com/datasets/m4vr79wsjs).

Human research participants

Policy information about studies involving human research participants and Sex and Gender in Research.

Reporting on sex and gender	There are no information on sex and gender of the fetal samples, which are anonymous.
Population characteristics	Fetal intestinal tissues were obtained from elective abortions with informed consent.<br>The gestational age ranged from 14 to 22 weeks. The gender of fetus is unknown.
Recruitment	The patients who did elective abortions in the Contraception, Abortion and Sexuality (CASA) in Leiden and The Hague were included after informed consent. The gestational age of the fetuses ranged from 14 to 22 weeks.
Ethics oversight	The work described here was reviewed and approved by the Medical Ethical Committee of Leiden University Medical Centre (P08.087). All experiments were conducted in accordance with local ethical guidelines and the principles of the Declaration of Helsinki.

Note that full information on the approval of the study protocol must also be provided in the manuscript.

Field-specific reporting

Please select the one below that is the best fit for your research. If you are not sure, read the appropriate sections before making your selection.

☒ Life sciences ☐ Behavioural & social sciences ☐ Ecological, evolutionary & environmental sciences

For a reference copy of the document with all sections, see nature.com/documents/nr-reporting-summary-flat.pdf

Life sciences study design

All studies must disclose on these points even when the disclosure is negative.

Sample size	In this study no statistical methods were used to predetermine sample size and a total of ~50 unique human fetal intestinal samples were used. For the high-dimensional single-cell assays the number was chosen to obtain sufficient number of immune cells to capture the heterogeneity. For the cell proliferation assays and functional profile studies, replicates were included to reproduce findings in independent experiments. All replication attempts were successful. See Figure legends for these specifications. In addition, 8 fetal intestinal samples were used for imaging mass cytometry and RNAscope analysis to investigate the immune composition and lymphoid follicles. 28 fetal, 2 pediatric and 6 adult samples were analyzed by single cell spectral flow cytometry to compare immune composition.
Data exclusions	In accordance with current practice in the field, single cells which failed standard QC for flow cytometry in OMIQ were excluded from analysis.
Replication	All attempts at replication were successful. And at least 3 independent experiments were performed for every finding.
Randomization	All experiments were performed using randomly selected human fetal intestinal samples with different gestational weeks, non-inflamed intestinal samples from pediatric and adult patients.
Blinding	No blinding test in this study. Since all the samples were selected and grouped randomly and data collection and /or analysis were mainly performed using computational tool, we consider that blinding was not applicable to the current study.

Reporting for specific materials, systems and methods

We require information from authors about some types of materials, experimental systems and methods used in many studies. Here, indicate whether each material, system or method listed is relevant to your study. If you are not sure if a list item applies to your research, read the appropriate section before selecting a response.

Materials & experimental systems

n/a	Involved in the study
☐	☒ Antibodies
☒	☐ Eukaryotic cell lines
☒	☐ Palaeontology and archaeology
☒	☐ Animals and other organisms
☒	☐ Clinical data
☒	☐ Dual use research of concern

Methods

n/a	Involved in the study
☒	☐ ChIP-seq
☐	☒ Flow cytometry
☒	☐ MRI-based neuroimaging

Antibodies

Antibodies used

Antibodies for flow cytometry immunophenotypic studies:
Antigen Tag Clone Supplier Cat. Dilution
1 CCR6 BB700 11A9 BD Biosciences 566478 40
2 CCR7 Spark NIR 685 G043H7 Biolegend 353257 25
3 CD117 VioBright 515 REA787 MACS 130-111-674 200
4 CD11c PE/Cy7 3.9 Biolegend 301608 100
5 CD127 BV711 A019D5 Biolegend 351327 25
6 CD161 BUV563 HP-3G10 BD Biosciences 749223 25
7 CD163 BV650 GHI/61 BD Biosciences 563888 25
8 CD1c SB436 L161 Invitrogen 62-0015-41 25
9 CD20 BV805 2H7 BD Biosciences 612906 50
10 CD25 BV421 BC96 Biolegend 302630 50
11 CD3 BV510 UCHT1 Biolegend 300448 100
12 CD34 PE/CF594 581 BD Biosciences 562383 50
13 CD4 CF568 SK3 CyTek R7-20041 200
14 CD45 QD800 HI30 Invitrogen Q10156 100
15 CD45R0 BV570 UCHL1 Biolegend 304225 200
16 CD45RA PerCP HI100 Biolegend 304155 200
17 CD69 APC/R700 FN50 BD Biosciences 565155 50
18 CD7 BUV395 M-T701 BD Biosciences 565979 100
19 CD8a Spark Blue 550 SK1 Biolegend 344759 200
20 CD8b BUV496 2ST8.5H7 BD Biosciences 749837 200
21 FoxP3 APC PCH101 Invitrogen 17-4776-42 25
22 HLA-DR BV480 G46-6 BD Biosciences 566154 200
23 Ki67 BV605 Ki-67 Biolegend 350521 25
24 RORgt PE Q21-559 BD Biosciences 563081 25
25 Viability L/D near IR - Invitrogen L34976 1000
26 FC Block - - Biolegend 422302 20

Imaging-mass cytometry antibody panel:
Antigen Tag Clone Supplier Cat. Dilution
1 CD45 89Y HI30 Flui 3089003B 50
2 D2-40 115In D2-40 BioL 916606 50
3 FOXp3 142Nd D608R CST 12653BF 100
4 CD69 144Nd FN50 Flui 3144018B 50
5 CD4 145Nd RPA-T4 Flui 3145001B 50
6 CD8a 146Nd RPA-T8 Flui 3146001B 50
7 Collagen I 147Sm polyclonal Millipore AB758 100
8 CD34 148Nd QBEND/10 Thermo MA1-10202 50
9 CD31 149Sm 8 9C2 CST CST3528BF 100
10 E-cadherin 150Nd 24 E 10 CST CST 3195BF 50
11 CD123 151Eu 6H6 Flui 3151001B 50
12 CD141 152Sm Phx-01 BioL 902102 50
13 CD7 153Eu CD7-6B7 Flui 3153014B 100
14 CD163 154Sm GHI/61 Flui 3154007B 100
15 CD103 155Gd EPR4166 Abcam ab221210 50
16 CD127 156Gd R34.34 Beckman 18LIQ494 50
17 CD68 159Tb KP1 Flui 3159035D 200
18 CD20 161Dy H1 Flui 3161029D 50
19 CD11c 162Dy Bu15 Flui 3162005B 50
20 CD11c 162Dy S-HCL-3 BioL 125602 50
21 CD161 164Dy HP-3G10 Flui 3164009B 50
22 CD117 165Ho 104D2 BioL 313202 50
23 Ki-67 166Er D3B5 CST CST 9129BF 200
24 CD27 167Er O323 Flui 3167002B 50
25 HLA-DR 168Er L243 BIoL 307651 800
26 CD45RA 169Tm HI100 Flui 3169008B 100
27 CD3 170Er UCHT1 Flui 3170001B 100
28 CD1c 171Yb L161 BioL 331501 50

29 CD38 172Yb HIT2 Flui 3172007B 100
30 CD45RO 173Yb UCHL1 BioL 304239 50
31 CD57 174Yb HNK-1/Leu-7 Abcam Ab212403 100
32 CD25 175Lu 24204.0 Thermo MA5-23714 50
33 CD56 176Yb NCAM16.2 Thermo MA1-06801 50
34 α-SMA 194Pt 1A4 CST 56856BF 100
35 Vimentin 198Pt D21H3 CST 5741BF 100

The antibody panel for cell proliferation assays:
Antigen Tag Clone Supplier Cat. Dilution
1 CD117 VioBright 515 REA787 MACS 130-111-674 200
2 CD11c PE/Cy7 3.9 Biolegend 301608 100
3 CD127 BV711 A019D5 Biolegend 351327 25
4 CD161 BUV563 HP-3G10 BD Biosciences 749223 25
5 CD20 BUV805 2H7 BD Biosciences 612906 50
6 CD25 BV421 BC96 Biolegend 302630 50
7 CD3 BV510 UCHT1 Biolegend 300448 100
8 CD4 BUV661 SK3 BD Biosciences 612962 100
9 CD45 NovaBlue 610 2D1 Invitrogen H005T02B05 50
10 CD7 BUV395 M-T701 BD Biosciences 565979 100
11 CD8a SB550 SK1 Biolegend 344759 200
12 CD8b BUV496 2ST8.5H7 BD Biosciences 749837 200
13 HLA-DR BV750 L243 Biolegend 307671 50
14 Ki67 R718 B56 BD Biosciences 566963 100
15 Viability L/D near IR - Invitrogen L34976 1000
16 CellTrace™ Violet CTV - Invitrogen 34557 4000
17 FC Block - - Biolegend 422302 20

The antibody panel for functional profile analysis:
Antigen Tag Clone Supplier Cat. Dilution
1 CD117 VioBright 515 REA787 MACS 130-111-674 200
2 CCR7 Spark NIR 685 G043H7 Biolegend 353257 25
3 CD127 BV711 A019D5 Biolegend 351327 25
4 CD161 BUV563 HP-3G10 BD Biosciences 749223 25
5 CD20 BUV805 2H7 BD Biosciences 612906 50
6 CD25 BV421 BC96 Biolegend 302630 50
7 CD3 BV510 UCHT1 Biolegend 300448 100
8 CD4 BUV661 SK3 BD Biosciences 612962 100
9 CD45 NovaBlue 610 2D1 Invitrogen H005T02B05 50
10 CD7 BUV395 M-T701 BD Biosciences 565979 100
11 CD8a SB550 SK1 Biolegend 344759 200
12 CD8b BUV496 2ST8.5H7 BD Biosciences 749837 200
13 CD45R0 BV570 UCHL1 Biolegend 304225 200
14 CD45RA PerCP HI100 Biolegend 304155 200
15 HLA-DR BV750 L243 Biolegend 307671 50
16 Ki67 BV605 Ki-67 Biolegend 350521 25
17 Helios PerCP/eFluor710 22F6 Thermo 46-9883-42 25
18 IL-17A PE/Dazzle™ 594 BL168 Biolegend 512335 25
19 IFNg BV750 RUO BD Biosciences 566357 50
20 TNFa PE/Cy7 MAb11 Thermo 25-7349-82 50
21 CD40L PE/Cy5 24-31 Biolegend 310808 50
22 Granzyme B R718 B56 BD Biosciences 566963 50
23 IL-2 PE MQ1-17H12 BD Biosciences 560902 200
24 Viability L/D near IR - Invitrogen L34976 1000
25 CellTrace™ Violet CTV - Invitrogen 34557 4000
26 FC Block - - Biolegend 422302 20

Validation

All antibodies used in flow cytometry were verified by staining human peripheral blood mononuclear cell and/or tonsil cells with known expression or not. Self-conjugation antibodies with metal used in imaging mass cytometry were validated on frozen intestinal tissue by IHC before conjugation, and pre-conjugation antibodies were purchased from Fluidigm.

Flow Cytometry

Plots

Confirm that:

☒ The axis labels state the marker and fluorochrome used (e.g. CD4-FITC).

☒ The axis scales are clearly visible. Include numbers along axes only for bottom left plot of group (a 'group' is an analysis of identical markers).

☒ All plots are contour plots with outliers or pseudocolor plots.

☒ A numerical value for number of cells or percentage (with statistics) is provided.

Methodology

Sample preparation	All the immune cells were isolated from human fetal intestines and stored in liquid nitrogen. Before staining, the cells were thawed and stained with specific panel as described in Methods:<br>The 26-antibody flow cytometry-based panel was developed for in-depth immunophenotyping of the major cell subsets present in the human fetal intestine through time. In total, 3 experiments were performed for immunophenotypic studies of 28 human fetal intestinal samples. Antibodies used for spectral flow cytometry with a 5-laser Cytek® Aurora are listed in Supplementary Table 1. For surface staining, single-cell suspensions of fetal intestinal samples were incubated with fluorochrome-conjugated antibodies and human Fc block (BioLegend) for 30 min at 4 °C. After washing, cells of samples were then fixed/permeabilized using Foxp3 Staining Buffer Set, according to manufacturer's instructions (ThermoFisher). For intracellular staining, the fixed/permeabilized cells were incubated with the antibodies for 45 min at 4 °C, followed by washing of the cells with permeabilization buffer. Then the stained cells were resuspended. Reference samples were incorporated and individually stained by UltraComp eBeadsTM Compensation Beads (ThermoFisher), PBMCs or cells from human tonsils. After the completion of the sample preparation, the samples were immediately acquired using a 5-laser Cytek® Aurora (BD Biosciences). Data were analyzed to check quality with FlowJo software version 10.6 (Tree Star Inc). We utilized OMIQ to perform the high-dimensional analysis for human fetal intestinal samples (https://www.omiq.ai/).
Instrument	The 5-laser Cytek® Aurora (BD Biosciences); Hyperion Imaging System (Fluidigm)
Software	OMIQ (https://www.omiq.ai/); FlowJo software version 10.6 (Tree Star Inc); Cytosplore Imaging (https://sec.lumc.nl/mtg-viewer/imaging/win/se_3.3.2/Cytosplore_Imaging_SE_3.3.2.zip)
Cell population abundance	All cell populations were distinct and purity was 95%-99%.
Gating strategy	All acquired cells were first gated on FCS/SSC and FCS/FCH, then L/D dye to obtain the single live cells. Every Flow cytometry experiment, a peripheral blood mononuclear cell control sample was included as staining and gating control. Based on the unstained sample and controls to set the positive and negative gates.

☒ Tick this box to confirm that a figure exemplifying the gating strategy is provided in the Supplementary Information.
